# Supplementary material for: Acupuncture for constipation in patients with stroke: protocol of a systematic review and meta-analysis
Source: BMJ Open. 2018 Mar 30;8(3):e020400. doi: 10.1136/bmjopen-2017-020400 (PMC5884333; doi:10.1136/bmjopen-2017-020400)
Supplement: Supplementary file 1 [file bmjopen-2017-020400supp001.pdf]

Appendix 1.

Pubmed Search strategy:

1. Cerebrovascular Disorders/
2. Exp Basal Ganglia Cerebrovascular Disease/
3. Exp Brain Ischemia/
4. Exp Carotid Artery Diseases/
5. Exp Cerebrovascular Trauma/
6. Exp Intracranial Arteriovenous Malformations/
7. Exp Intracranial Arterial Diseases/
8. Exp Intracranial Embolism and Thrombosis/
9. Exp Intracranial Hemorrhages
10. Stroke/
11. Exp Brain Infarction/
12. Stroke, Lacunar/
13. Vasospasm, Intracranial
14. Vertebral Artery Dissection/
15. Exp Hypoxia, Brain/
16. (stroke* OR post stroke OR poststroke OR post-stroke OR apoplex* OR cerebrovasc* OR CVA OR SAH OR cerebral vasc*).tw
17. ((brain OR cerebr* OR cerebell* OR vertebrobasil* OR hemispher* OR intracran* OR intracerebral OR infratentorial OR supratentorial OR middle cerebr* OR mca* OR anterior circulation OR basilar artery OR vertebral artery) AND (Ischemi* OR infarct*OR thrombos*OR thromboem* OR emboli*OR occlus* OR hypoxi*)).tw
18. ((Brain* OR cerebr* OR cerebell* OR intracerebral OR intracran* OR parenchymal OR intraparenchymal OR intraventricular OR infratentorial OR supratentorial OR basal gangli* OR putaminal OR putamen OR posterior fossa OR hemisphere* OR subarachnoid)) AND (haemorrhag* OR hemorrhag* OR haematoma* OR hematoma* OR bleed*)) .tw
19. Exp Hemiplegia/
20. Exp Paresis/
21. Exp Aphasia/
22. Exp Gait Disorders, Neurologic/
23. (Hemipar* OR hemipleg* OR paresis OR paretic OR aphasi* OR dysphasi*).tw
24. Exp Brain Damage, Chronic”/
25. Brain Injuries/
26. Exp Brain Concussion/
27. Exp Brain Hemorrhage,Traumatic/
28. Brain Injury, Chronic/
29. Diffuse Axonal Injury/
30. Craniocerebral Trauma/
31. Exp Head Injuries, Closed/
32. Exp Intracranial Hemorrhage, Traumatic/
33. Exp Brain Abscess/

34. Exp Central Nervous System Infections/
35. Exp Encephalitis/
36. Exp Meningitis/
37. (encephalitis OR meningitis OR head injur*).tw
38. Exp Brain Neoplasms/
39. ((brain OR cerebr*) AND (injur* OR hypoxi* OR damage* OR concussion OR trauma* OR neoplasm* OR lesion* OR tumor* OR tumour* OR cancer* OR infection)).tw
40. OR/ 1- 39
41. Constipation/
42. (Impaction OR obstipation OR costiveness OR defecation OR evacuation).tw
43. delayed bowel movement.tw
44. (bowel AND (function* OR habit* OR movement* OR symptom* OR motility OR stool*)).tw
45. colon transit.tw
46. intestinal motility.tw
47. OR/ 41-46
48. 40 AND 47
49. acupuncture/
50. exp acupuncture therapy/
51. electroacupuncture/
52. meridians/
53. acupuncture points/
54. acupunctur*.tw.
55. (electroacupuncture OR electro-acupuncture).tw.
56. acupoints.tw.
57. ((meridian OR non-meridian OR trigger) AND point*).tw.
58. OR/49-57
59. randomized controlled trial.pt
60. controlled clinical trial.pt
61. randomized.tw
62. placebo.tw
63. clinical trials as topic/
64. randomly .tw
65. trial.tw
66. OR/59-65
67. Animals/ NOT humans/
68. 65 NOT 67
69. 48 AND 58 AND 68

CBM Search strategy:

#1 中风 OR 卒中 OR 脑*塞 OR 脑*血 OR 脑*栓 OR 蛛网膜下腔出血
#2 主题词=中风/全部副主题词
#3 主题词=卒中/全部副主题词

#4 主题词=梗塞，大脑前动脉/全部副主题词
#5 主题词=梗塞，大脑中动脉/全部副主题词
#6 主题词=梗塞，大脑后动脉/全部副主题词
#7 主题词=蛛网膜下腔出血/全部副主题词
#8 #1~#7/OR
#9 随机 OR 盲法 OR 安慰剂
#10 主题词=随机对照试验[文献类型]
#11 主题词=随机分配
#12 主题词=随机对照试验/全部副主题词
#13 #9~#12/OR
#14 针刺 OR 电针 OR 火针 OR 头针 OR 毫针 OR 手捻针 OR 芒针 OR 巨针 OR 体针 OR 温针 OR 针灸
#15 主题词=针刺/全部副主题词
#16 主题词=针刺穴位/全部副主题词
#17 主题词=针刺疗法/全部副主题词
#18 主题词=针灸疗法/全部副主题词
#19 #14~#18/OR
#20 便秘 OR 排便 OR 腹胀 OR 腹痛
#21 主题词=便秘/全部副主题词
#22 主题词=排便/全部副主题词
#23 #20~#22/OR
#24 #8 AND #13 AND #23

CNKI Search strategy:
(SU=中风 OR SU=卒中 OR SU=脑梗 OR SU=脑栓塞 OR SU=脑出血 OR SU=脑血栓 OR SU=蛛网膜下腔出血) AND (SU=针刺 OR SU=电针 OR SU=火针 OR SU=头针 OR SU=毫针 OR SU=手捻针 OR SU=芒针 OR SU=巨针 OR SU=体针 OR SU=温针 OR SU=针灸) AND (SU=便秘 OR SU=排便 OR SU=腹胀 OR SU=腹痛) AND (SU=随机 OR FT=随机)

Wanfang Search strategy:
主题:(中风+卒中+脑梗+脑栓塞+脑出血+脑血栓+蛛网膜下腔出血)*主题:(针刺+电针+火针+头针+毫针+手捻针+芒针+巨针+体针+温针+针灸)*主题:(便秘+排便+腹胀+腹痛)*随机

Vip Search strategy:
M=(中风+卒中+脑梗+脑栓塞+脑出血+脑血栓+蛛网膜下腔出血)*M=(针刺+电针+火针+头针+毫针+手捻针+芒针+巨针+体针+温针+针灸)*M=(便秘+排便+腹胀+腹痛)*U=随机
