# Supplementary material for: Acupuncture for constipation in patients with stroke: protocol of a systematic review and meta-analysis
Source: BMJ Open. 2018 Mar 30;8(3):e020400. doi: 10.1136/bmjopen-2017-020400 (PMC5884333; doi:10.1136/bmjopen-2017-020400)
Supplement: Author's manuscript [file bmjopen-2017-020400.draft_revisions.pdf]

BMJ Open

BMJ Open is committed to open peer review. As part of this commitment we make the peer review history of every article we publish publicly available.

When an article is published we post the peer reviewers' comments and the authors' responses online. We also post the versions of the paper that were used during peer review. These are the versions that the peer review comments apply to.

The versions of the paper that follow are the versions that were submitted during the peer review process. They are not the versions of record or the final published versions. They should not be cited or distributed as the published version of this manuscript.

BMJ Open is an open access journal and the full, final, typeset and author-corrected version of record of the manuscript is available on our site with no access controls, subscription charges or pay-per-view fees (http://bmjopen.bmj.com).

If you have any questions on BMJ Open's open peer review process please email info.bmjopen@bmj.com

Acupuncture for constipation in stroke patients: protocol of a systematic review and meta-analysis

Journal:	*BMJ Open*
Manuscript ID	bmjopen-2017-020400
Article Type:	Protocol
Date Submitted by the Author:	02-Nov-2017

Complete List of Authors:	Zhai, Jingbo; Tianjin University of Traditional Chinese Medicine, Research institute of Traditional Chinese Medicine<br>Mu, Wei; The Second Affiliated Hospital of Tianjin University of Traditional Chinese Medicine, Department of Clinical Pharmacology<br>Si, Jinhua; Tianjin University of Traditional Chinese Medicine, Library<br>Li, Yan; Tianjin University of Traditional Chinese Medicine, School of Nursing<br>zhao, chen; Hong Kong Baptist University, Hong Kong Chinese Medicine Clinical study Centre<br>Shang, Hongcai; Dongzhimen Hospital, Beijing University of Chinese Medicine, Key Laboratory of Chinese Internal Medicine of Ministry of Education and Beijing<br>Li, Huanan; First Teaching Hospital of Tianjin University of Traditional Chinese Medicine, Laboratory for Biological Effects of Tuina<br>Tian, Guihua; Dongzhimen Hospital, Beijing University of Chinese Medicine, Key Laboratory of Chinese Internal Medicine of Ministry of Education and Beijing

Keywords:	Stroke medicine < INTERNAL MEDICINE, constipation, Acupuncture, systematic review, meta-analysis

SCHOLARONE™ Manuscripts

Acupuncture for constipation in stroke patients: protocol of a systematic review and meta-analysis

Jingbo Zhai[1], Wei Mu[2], Jinhua Si[3], Yan Li[4], Chen Zhao[5], Hongcai Shang[6], Huanan Li[7,8]*, Guihua Tian[6*]

[1]Research institute of Traditional Chinese Medicine, Tianjin University of Traditional Chinese Medicine, 312 Anshanxi Road, Nankai District, Tianjin 300193, China;

[2]Department of Clinical Pharmacology, The Second Affiliated Hospital of Tianjin University of Traditional Chinese Medicine, 69 Zengchan Road, Hebei District, Tianjin 300250, China;

[3]Library of Tianjin University of Traditional Chinese Medicine, 312 Anshanxi Road, Nankai District, Tianjin 300193, China;

[4]School of Nursing, Tianjin University of Traditional Chinese Medicine, 312 Anshanxi Road, Nankai District, Tianjin 300193, China;

[5]Hong Kong Chinese Medicine Clinical study Centre, Hong Kong Baptist University, 7 Baptist University Road, Kowloon Tong, Hong Kong, China;

[6]Key Laboratory of Chinese Internal Medicine of Ministry of Education and Beijing, Dongzhimen Hospital, Beijing University of Chinese Medicine, Beijing 100700, China;

[7]First Teaching Hospital of Tianjin University of Traditional Chinese Medicine, 314 Anshanxi Road, Nankai District, Tianjin 300193, China;

[8]Laboratory for Biological Effects of Tuina, State Administration of Traditional Chinese Medicine, 314 Anshanxi Road, Nankai District, Tianjin 300193, China;

Correspondence to Huanan Li, lihuanan1984@126.com; Guihua Tian, Rosetgh@163.com

ABSTRACT

Introduction:

Constipation is one of the most common complications in patients with stroke. Acupuncture has gained increased popularity for the management of constipation. However, there is a lack of supportive evidence on the efficacy of acupuncture for post-stroke constipation. This systematic review aims to collect and critically appraise all the available evidence about the efficacy and safety of the acupuncture for

For peer review only - http://bmjopen.bmj.com/site/about/guidelines.xhtml

constipation in post-stroke patients.

Methods and analysis:

A comprehensive search of Pubmed, Embase, Cochrane Central Register of Controlled Trials, Web of science, CNKI, CBM, WANFANG, and VIP database will be conducted to identify randomized controlled trials of acupuncture for constipation in post-stroke patients. There is no restriction on language or publication status.

The primary outcome measure will be frequency of bowel movement. The risk of bias will be assessed using the approach recommended by Cochrane Handbook for Systematic Reviews of Interventions.

We will conduct the meta-analysis to synthesise the evidence for each outcome, if possible. The heterogeneity will be statistically assessed using a Chi^2 test and I^2 statistic.

This protocol is developed following the guideline of PRISMA-P 2015.

Ethics and dissemination:

The ethical approval is not required because no primary data is collected. The findings will be presented at scientific conferences or a peer-reviewed scientific journal.

This protocol has been registered on PROSPERO (CRD42017076880).

Strengths and limitations of this study

- This review will provide a comprehensive assessment regarding the effect of acupuncture for constipation in patients with stroke.
- Only randomized controlled trials will be included which are more likely to provide unbiased information than other study designs.
- PRISMA-P guideline for meta-analysis protocols is followed.
- GRADE approach will used to assess the quality of the body of evidence.
- This review will be fundamental for reliable recommendations in the management of post-stroke constipation.

Introduction

Stroke is one of the leading causes of death and disability worldwide[1]. The incidence of stroke is subject to large variation globally[2]. The overall incidence in low to middle income countries exceeded that in high-income countries by 20% from 2000 to 2008[2].

Constipation is one of the most common complications in patients with stroke[3]. The evidence from a previous systematic review shows that the incidence of constipation in stroke patients is 48% (95% confidence interval, 33% to 63%)[4]. The incidence of constipation in patients with hemorrhagic stroke is higher than that in patients with ischemic type[4]. The incidence in rehabilitation stage is higher than that in acute stage[4].

Constipation has a negative impact on the patient's psycholigical well-being and physical health, restricts social activities, reduces quality of life, contributes to a poor

For peer review only - http://bmjopen.bmj.com/site/about/guidelines.xhtml

outcome and is associated with high healthcare costs[4-6].

Stool softeners, prokinetic agents, osmotic and stimulant laxatives, and lifestyle or dietary modification are common treatments for constipation[7]. Conventional treatments may be associated with unwanted side effects, such as bloating, dehydration, a high recurrence rate after ceasing drugs[7,8]. Most of patients with chronic constipation are not satisfied with current treatment options in Europe[9]. Many constipation patients seek help from alternative therapies[9].

Acupuncture, as an important part of complementary and alternative medicine (CAM), has gained increased popularity for the management of constipation in Western countries[9]. There are some possible mechanisms of acupuncture for constipation[8]. Acupuncture can promote intestinal canal peristalsis through contacting the intestinal wall, and regulating nervous and body fluids[8]. Acupuncture can increase rectal internal pressure to restrore the defecation sense by stimulating parasympathetic nerve[8].

A systematic review shows that acupuncture is safe for chronic functional constipation[10]. A randomized trial supports the use of electroacupuncture for chronic severe functional constipation[11]. However, whether the evidence is transferrable to the stroke population remains unclear.

The recent studies mainly focus on the incidence of constipation after stroke[4,5]. The management strategies for constipation in stroke patients remains poorly investigated[3].

A systematic review evaluated the efficacy and safety of acupuncture and moxibustion for post-stroke constipation in 2014[12]. This review had some obvious flaws that might threaten the authenticity of their findings. First, the meta-analysis was not conducted to assess the efficacy of acupuncture and moxibustion, respectively. The efficacy of acupuncture alone for constipation in stroke patients was not systematically assessed. Second, the methodological quality of eight included articles was very poor. Third, the total effective rate, a subjective outcome measure, was chosed as the primary outcome. Forth, the sample size was small. To our knowledge, several new RCTs have been published since the meta-analysis was published[13,14]. Overall, there is a lack of supportive evidence on the efficacy and safety of acupuncture for constipation in patients with stroke.

The aim of this study is to systematically review current available literature to assess the efficacy and safety of the acupuncture treatment for post-stroke constipation.

Methods:

This protocol is developed following the guideline of PRISMA-P 2015[15].

Inclusion criteria

Types of studies

We will only include randomized controlled trials (RCTs) which are more likely to provide unbiased information than other study designs[16]. We will exclude quasi-randomized RCTs, such as those allocating by alphabetical order, alternate days

For peer review only - http://bmjopen.bmj.com/site/about/guidelines.xhtml

of the week, or date of birth. Cross-over trials will be excluded because of potential for a carry-over effect. There is no restriction on language or publication status.

Types of participants

We will include adults (over 18 years old) suffering from constipation after a first or recurrent stroke. Trials will be excluded in which patients have a prior history of constipation before the stroke diagnosis. Stroke is defined as 'rapidly developed signs of focal or global disturbance of cerebral funcation, lasting more than 24 hours or leading to death with no apparent cause other than that of vascular origin' according to WHO criteria[17]. We will include stroke patients irrespective of any type (ischaemic or haemorrhagic) or phase (acute, subacute or chronic). Acute and subacute stroke is defined as less than six months since onset, and chronic stroke lasts more than six months since onset[18].

All of patients should be diagnosed as constipation according to at least one of the current or past definitions or guidelines of constipation, such as Rome □/□ diagnostic criteria or guidelines for clinical research on Chinese new herbal medicine[12].

There is no restriction on age, sex or ethnicity of the enrolled subjects.

Types of interventions

Experimental interventions

We will include trials using either traditional or contemporary acupuncture. Traditional acupuncture refers to needles inserted in classical meridian points[19]. Contemporary acupuncture refers to needles inserted in non-meridian or trigger points regardless of the source of stimulation (for example, hand, electrical stimulation or fine needle) [19]. We will exclude trials in which treatment without needling, such as acupressure, tap-pricking, point injection, laser acupuncture. No restrictions are imposed on times of treatment and length of treatment period.

Comparator interventions

The control interventions could be placebo acupuncture, sham acupuncture, no treatment, another active treatment or medication.

Placebo acupuncture refers to a needle attached to the skin surface without penetrating the skin[20].

Sham acupuncture is defined as a needle placed in an area close to but not in acupuncture points or subliminal skin electrostimulation via electrodes attached to the skin[20].

We consider another active treatment or medication to be pharmacologic and nonpharmacologic treatment or medication, such as laxatives, emollients, kubricants, lifestyle or dietary modification.

We will investigate the comparisons listed below:

1. acupuncture only compared with no treatment;
2. acupuncture only compared with placebo or sham treatment;
3. acupuncture plus another active treament or medication compared with another

For peer review only

For peer review only - http://bmjopen.bmj.com/site/about/guidelines.xhtml

active treament or medication alone;
4. acupuncture plus another active treament or medication compared with placebo or sham treatment plus another active treament or medication.

Types of outcome measures:

Primary outcome

The primary outcome measure will be frequency of bowel movement[21,22]. Bowel movement frequency is defined as the mean number of spontaneous bowel movements per week[23].

Secondary outcome

Secondary outcome measures include proportion of patients with stool consistency, proportion of patients using rescue medication such as laxatives or rectal evacuants, Quality of life (QoL), and mean transit time.

Stool consistency will be defined by trialists or measured based on scales such as Bristol Stool Form Scale (BSFS) [24].

QoL will be measured by generic or condition-specific scales, such as Short Form 36 Health Surveys (SF-36)[25].

Transit time is defined as the time from the first perception of wanting to defaecate to the finish of defaecation[26].

We will sum up the number of adverse events (AEs), and calculate the proportion of AEs[23].

Search strategy

Electronic searches

The published literature will be identified by searching Pubmed, Embase, Cochrane Central Register of Controlled Trials, and Web of science. CNKI, CBM, WANFANG, and VIP database will also be systematically searched to identify any relevant study published in China.

The search strategy is developed by a medical librarian (JS) according to key terms from previous literature reviews[27,28]. The detailed search strategy for the pubmed database is attached (appendix 1). The terms will be modified as necessary for other databases. We will not apply any language or date restrictions.

Searching other resources

We will check the reference lists of identified relevant RCTs and reviewed articles for additional studies. We will contact experts in the field of stroke and constipation to identify any additional trials.

The World Health Organization International Clincial trials Registry platform (ICTRP), ClinicalTrials.gov will also be checked to identify planned, ongoing or unpublished trials. Google Scholar will be searched to identify any grey literature.

For peer review only - http://bmjopen.bmj.com/site/about/guidelines.xhtml

Data collection and analysis

Selection of studies

Two review authors (WM and JS) will independently assess abstracts and titles of studies identified by literature search. Duplicates will be omitted using EndNote software (version X7.0)[29]. Revelant studies will be selected against the predefined inclusion criteria. If necessary, reviewers will examine full-text reports to identify eligible studies. EndNote software will also be used to manage records. We will illustrate the selection process in a PRISMA diagram[30]. Any disagreement will be resolved by consensus.

Data extraction and management

Two review authors (YL and CZ) will independently extract data from the included studies. Calibration exercises will be conducted to ensure consistency across reviewers before starting the review. The following information will be extracted using a predetermined data form: general information (title, authors, country of study, funding, year of publication, registry number); details of study (aim, design, inclusion and exclusion criteria, method of randomization and allocation); study population (age, sex, sample size, number for analysis, type of stroke); intervention characteristics (type, duration, dose, follow-up time points, compliance); outcome (primary and secondary outcomes, time points, method of outcome assessments, blinding of outcome assessment, adverse effects).

Where necessary we will contact study authors for clarification of data and additional information. Any disagreement will be resolved by consensus or consultation with a third review author.

Assessment of risk of bias in included studies

Two authors (HL and HS) will independently assess the risk of bias using the approach recommended by Cochrane Handbook for Systematic Reviews of Interventions[31]. The following risk of bias domains will be assessed: sequence generation (selection bias); allocation concealment (selection bias); blinding of participants and personnel (performance bias); blinding of outcome assessment (detection bias); incomplete outcome data (attrition bias); selective outcome reporting (reporting bias); other bias.

We will attempt to describe what is reported to have happened in each study for each domain of risk of bias. Thus, we will be able to provide the rationale for the judgement of whether this domain is at low, high, or unclear risk of bias. Where necessary we will contact authors of included studies for missing information or clarification.

If all domains are at low risk of bias, the overall risk of bias of individual studies will be categorised as low risk of bias. Otherwise, overall risk of bias will categorised as high risk of bias[32]. The ‘Risk of bias’ summary will be presented graphically.

For peer review only - http://bmjopen.bmj.com/site/about/guidelines.xhtml

Measures of treatment effect

We will use the risk ratio (RR) with 95% confidence intervals (CI) to express the estimate of effect for dichotomous outcome.

For continuous outcomes, we will express the estimate of effect as mean difference (MD) with 95% CI. When the same outcome is measured in a variety of ways, the standardized mean difference (SMD) with 95% CI will be used to express the size of the intervention effect.

Dealing with missing data

We will attempt to contact study authors for missing data or clarification, where feasible. The following strategies will be used to evaluate the potential influence of missing data[32].

1. Worst-case scenario analysis: all participants with missing data counted as failures.
2. Extreme worst-case analysis: participants with missing data in experimental group counted as failures and participants with missing data in control group counted as successes.
3. Extreme best-case analysis: participants with missing data in experimental group counted as successes and participants with missing data in control group counted as failures.

Assessment of heterogeneity

We will assess the statistical heterogeneity using a Chi^2 test[33]. In addition, we will quantify heterogeneity using the I^2 statisitc value which ranges from 0% to 100%[34]. $P < 0.1$ of Chi^2 test or $I^2 > 50\%$ indicates statistically significant heterogeneity[33,34]. Potential clinical heterogeneity will be assessed by prespecified subgroup analyses.

Assessment of reporting baises

When a meta-analysis includes 10 or more RCTs, we will assess asymmetry using funnel plots visually[32]. In addtition, we will test asymmetry using the Harbord modified test for dichotomous outcomes and Egger test for continuous outcomes[32].

Data synthesis

We will combine more than one trial to estimate pooled intervention effect using the meta-analysis when studies examine the same intervention and outcomes with comparable methods in similar populations[31].

We will pool the continuous data using the inverse variance method, and dichotomous data using the Mantel-Haenszel method[31].

We will use the fixed-effect model to combine data when statistical heterogeneity is low. However, when $P < 0.1$ or $I^2 > 50\%$, the random-effect model will be used to provide a more conservative estimate of effect[35].

All analyses will be conducted with Review Manager 5.3 software. If a meta-analysis is not possible, we will provide a narrative summary of the results from individual studies.

For peer review only - http://bmjopen.bmj.com/site/about/guidelines.xhtml

Subgroup analysis and investigation of heterogeneity

We will perform the following subgroup analyses to investigate heterogeneity when sufficient data are available. We will conduct subgroup analyses based on age, sex, type of stroke (hemorrhagic and ischemic stroke), different definitions of constipation, phase of stroke (acute, subacute or chronic), and type of control group (placebo, sham acupuncture, no treatment or another active treament or medication).

The intervention effect will be analyzed using the Chi^2 test, with $P < 0.05$ indicating statistically significant differences between subgroups.

Sensitivity analysis

We will carry out sensitivity analyses to evaluate the robustness of the pooled results excluding trials with high risk of bias, and the option of using missing data (worst-case scenario analysis, extreme worst-case analysis, or extreme best-case analysis) [32,34].

Summary of findings table

We will prepare 'Summary of findings' tables including a grade of the overall quality of the body of evidence for each outcome using GRADEpro GDT[36].

Two review authors will independently assess the quality of the body of evidence according to five GRADE criteria: study limitations, imprecision, inconsistency, indirectness, and publication bias. It will fall into one of four possible ratings (high, moderate, low and very low). Any discrepancy will be resolved by consensus or consultation with a third review author.

Amendments

We will provide the date of any amendment, a description of the change and the rationale in the event of protocol amendments.

Dissemination

This review will provide a comprehensive assessment regarding the effect of acupuncture for constipation in patients with stroke. The results will be fundamental for reliable recommendations in the management of post-stroke constipation.

We will present findings from this systematic review at scientific conferences and publish the findings in a peer-reviewed scientific journal accroding to the PRISMA guidelines.

Protocol registration: This protocol has been registered on PROSPERO (CRD42017076880).

Contributors:

JZ, HL and GT conceived the study. JZ, HL and GT provided general guidance to the drafting of the protocol. JZ and WM drafted the protocol. JS designed the search strategy. JZ, YL, CZ and HS drafted the manuscript. JZ, WM, JS, YL, CZ, HL, GT and HS reviewed and revised the manuscript. All authors have read and approved the final version of the manuscript.

For peer review only - http://bmjopen.bmj.com/site/about/guidelines.xhtml

Funding:

This study is supported by the National Natural Science Foundation of China [grant number 81703936] and Beijing Nova Program [grant number xx2014B049].

Competing interests: None declared.

Ethics approval: Not required.

Provenance and peer review: Not commissioned; extemallly peer reviewed.

Data sharing statement: We will publish findings from this systematic review in a peer-reviewed scientific journal, and data set will be made freely available.

Open access: This is an Open Access article distributed in accordance with the terms of the Creative Commons Attribution (CC BY 4.0) license, which permits others to distribute, remix, adapt and build upon this work, for commercial use, provided the original work is properly cited. See: http://creativecommons.org/licenses/by/4.0/

References:

[1] Kathleen Strong, Colin Mathers, Ruth Bonita. Preventing stroke: saving lives around the world. Lancet 2007;6:182-187.

[2] Valery L Feigin, Carlene M M Lawes, Derrick A Bennett, et al. Worldwide stroke incidence and early case fatality reported in 56 population-based studies: a systematic review. Lancet Neurol 2009;8(4):355-369.

[3] Su Fee Lim, Charmaine Childs. A systematic review of the effectiveness of bowel management strategies for constipation in adults with stroke. Int J Nurs Stud 2013;50(7):1004-1010.

[4] Jianxiang Li, Mengguo Yuan, Yunfang Liu, et al. Incidence of constipation in stroke patients: A systematic review and meta-analysis. Medicine 2017;96:e7225.

[5] Yongjing Su, Xiaoyan Zhang, Jinsheng Zeng, et al. New-Onset Constipation at Acute Stage After First Stroke Incidence, Risk Factors, and Impact on the Stroke Outcome. Stroke 2009;40(4):1304-1309.

[6] Umar Shariff, Omar M Aboumarzouk, Richard L Nelson. Antibiotics for the management of constipation predominant irritable bowel syndrome and chronic constipation. Cochrane Database Syst Rev 2009;(1):CD007518.

[7] Shengli Zhou, Xiulai Zhang, Jinghua Wang. Comparison of electroacupuncture and medical treatment for functional constipation: a systematic review and meta-analysis. Acupunct Med 2017;0:1-7.

[8] Chaoxian Zhang, Like Guo, Xiaofeng Guo, et al. Short and long-term efficacy of combining Fuzhengliqi mixture with acupuncture in treatment of functional constipation[J]. J Tradit Chin Med 2013;33(1):51-59.

[9] Tack J, Mueller-Lissner S, Dubois D, Schenck F. Only 27% of European patients

For peer review only - http://bmjopen.bmj.com/site/about/guidelines.xhtml

with chronic constipation are satisfied with current treatment options. Gut 2009; 58(Suppl □):A181.
[10] Tao Zhang, Tony Y. Chon, Baoyan Liu, et al. Efficacy of Acupuncture for Chronic Constipation: A Systematic Review. Am J Chin Med 2013;41(4):717-742.
[11] Zhishun Liu, Shiyan Yan, Jiani Wu, et al. Acupuncture for Chronic Severe Functional Constioation: A randomized trial. Ann Intern Med 2016;165(11):761-769.
[12] Yang Jipeng, Liu Jingying, Gu Hongyan, et al. Randomized controlled trials of acupuncture and moxibustion for post-stroke constipation: a meta analysis. Zhongguo Zhen Jiu 2014;34(8):833-836.
[13] Wang Chengwei, Liu Mengyue, Wen Qian, et al. Clinical observation of preventing and treating constipation by electroacupunture intervention in ischemic stroke patients at acute stage. Zhongguo Zhen Jiu 2015;35(5):430-434.
[14] Liu Haifei, Song Fengjun, Ye Bihong. Therapentic observation of acupuncture at He-Sea and Front-Mu points for post-stroke constipation. Shanghai J Acu-mox 2016;35(2):160-161.
[15] Larissa Shamseer, David Moher, Mike Clarke, et al. Preferred reporting items for systematic review and meta-analysis protocols (PRISMA-P) 2015: elaboration and explanation. BMJ 2015;349:g7647.
[16] Lise Lotte Gluud, Marsha Y Morgan. Endoscopic therapy and beta-blockers for secondary prevention in adults with cirrhosis and oesophageal varices. Cochrane Database Syst Rev 2017;6:CD012694.
[17] Hatano S. Experience from a multicentre stroke register: a preliminary report. Bull World Health Organ 1976;54(5):541–553.
[18] Ryo Momosaki, Naoki Yamada, Erika Ota, et al. Repetitive peripheral magnetic stimulation for activities of daily living and functional ability in people after stroke. Cochrane Database Syst Rev 2017;6:CD011968.
[19] Cheong YC, Dix S, Hung Yu Ng E, et al. Acupuncture and assisted reproductive technology. Cochrane Database Syst Rev 2013;7:CD006920.
[20] Daniel KL Cheuk, Virginia Wong, Wen Xiong Chen. Acupuncture for autism spectrum disorders. Cochrane Database Syst Rev 2011;9:CD007849.
[21] Sue Woodward, Christine Norton, Pauline Chiarelli. Biofeedback for treatment of chronic idiopathic constipation in adults. Cochrane Database Syst Rev 2014;3:CD008486.
[22] Eunice B Turawa, Alfred Musekiwa, Anke C Rohwer. Interventions for preventing postpartum constipation. Cochrane Database Syst Rev 2015;9:CD011625.
[23] Min Chen, Hui Zheng, Juan Li, et al. Non-pharmacological treatments for adult patients with functional constipation: a systematic review protocol. BMJ Open 2014;4(6):e004982.
[24] Larry E. Miller, Alvin lbarra, Arthur C. Ouwehand, et al. Normative values for stool frequency and form using Rome III diagnostic criteria for functional constipation in adults: systematic review with meta-analysis. Ann Gastroenterol 2017;30(2):161-167.
[25] Maureen Coggrave, Christine Norton, June D Cody. Management of faecal incontinence and constipation in adults with central neurological diseases. Cochrane

For peer review only

Database Syst Rev 2014;1:CD002115.
[26] Qianhua Zheng, Hui Zheng, Lingyun Lu, et al. Acupuncture for functional constipation: protocol of an individual patient data meta-analysis. BMJ Open 2015;5(5):e007137.
[27] Zhang S, Liu M, Asplund K, et al. Acupuncture for acute stroke. Cochrane Database Syst Rev 2005;2:CD003317.
[28] Omar M Aboumarzouk, Trisha Agarwal, Ramez Antakia, et al. Cisapride for Intestinal Constipation. Cochrane Database Syst Rev 2011;1:CD007780.
[29] Guomin Song, Xiaoling Liu, Wei Bian, et al. Systematic review with network meta-analysis: comparative efficacy of different enteral immunonutrition formulas in patients underwent gastrectomy. Oncotarget 2017;8(14):23376-23388.
[30] Alessandro Liberati, Douglas G. Altman, Jennifer Tetzlaff, et al. The PRISMA Statement for Reporting Systematic Reviews and Meta-Analyses of Studies That Evaluate Health Care Interventions: Explanation and Elaboration. Plos Med 2009; 6(7):e1000100.
[31] Higgins JPT, Green S, eds. Cochrane handbook for systematic reviews of interventions version 5.1.0. [updated March 2011]. The Cochrane Collaboration, 2011. http://www.handbook.cochrane.org.
[32] Luise Aamann, Gitte Dam, Anders Rinnov, et al. Physical exercise for people with cirrhosis. Cochrane Database Syst Rev 2017;6:CD012678.
[33] Robert P Anthonappa, Paul F Ashley, Debbie L Bonetti, et al. Non-pharmacological interventions for managing dental anxiety in children. Cochrane Database Syst Rev2017;6:CD012676.
[34] Boonstra A, van Dulmen-den Broeder E, Rovers MM, et al. Severe fatigue in childhood cancer survivors. Cochrane Database Syst Rev 2017;6:CD012681.
[35] Browning GG, Howell P, Whitmer WM, et al. Unilateral versus bilateral hearing aids for bilateral hearing impairment in adults. Cochrane Database Syst Rev 2017;5:CD012665.
[36] GRADEpro GDT. GRADEpro Guideline Development Tool. 2015. https://gradepro.org.

For peer review only

For peer review only - http://bmjopen.bmj.com/site/about/guidelines.xhtml

Appendix 1.

Pubmed Search strategy:

1. Cerebrovascular Disorders/
2. Exp Basal Ganglia Cerebrovascular Disease/
3. Exp Brain Ischemia/
4. Exp Carotid Artery Diseases/
5. Exp Cerebrovascular Trauma/
6. Exp Intracranial Arteriovenous Malformations/
7. Exp Intracranial Arterial Diseases/
8. Exp Intracranial Embolism and Thrombosis/
9. Exp Intracranial Hemorrhages
10. Stroke/
11. Exp Brain Infarction/
12. Stroke, Lacunar/
13. Vasospasm, Intracranial
14. Vertebral Artery Dissection/
15. Exp Hypoxia, Brain/
16. (stroke* OR post stroke OR poststroke OR post-stroke OR apoplex* OR cerebrovasc* OR CVA OR SAH OR cerebral vasc*).tw
17. ((brain OR cerebr* OR cerebell* OR vertebrobasil* OR hemispher* OR intracran* OR intracerebral OR infratentorial OR supratentorial OR middle cerebr* OR mca* OR anterior circulation OR basilar artery OR vertebral artery) AND (Ischemi* OR infarct*OR thrombos*OR thromboem* OR emboli*OR occlus* OR hypoxi*)).tw
18. ((Brain* OR cerebr* OR cerebell* OR intracerebral OR intracran* OR parenchymal OR intraparenchymal OR intraventricular OR infratentorial OR supratentorial OR basal gangli* OR putaminal OR putamen OR posterior fossa OR hemisphere* OR subarachnoid)) AND (haemorrhag* OR hemorrhag* OR haematoma* OR hematoma* OR bleed*)) .tw
19. Exp Hemiplegia/
20. Exp Paresis/
21. Exp Aphasia/
22. Exp Gait Disorders, Neurologic/
23. (Hemipar* OR hemipleg* OR paresis OR paretic OR aphasi* OR dysphasi*).tw
24. Exp Brain Damage, Chronic"/
25. Brain Injuries/
26. Exp Brain Concussion/
27. Exp Brain Hemorrhage,Traumatic/
28. Brain Injury, Chronic/
29. Diffuse Axonal Injury/
30. Craniocerebral Trauma/
31. Exp Head Injuries, Closed/
32. Exp Intracranial Hemorrhage, Traumatic/
33. Exp Brain Abscess/

For peer review only

34. Exp Central Nervous System Infections/
35. Exp Encephalitis/
36. Exp Meningitis/
37. (encephalitis OR meningitis OR head injur*).tw
38. Exp Brain Neoplasms/
39. ((brain OR cerebr*) AND (injur* OR hypoxi* OR damage* OR concussion OR trauma* OR neoplasm* OR lesion* OR tumor* OR tumour* OR cancer* OR infection)).tw
40. OR/ 1- 39
41. Constipation/
42. (Impaction OR obstipation OR costiveness OR defecation OR evacuation).tw
43. delayed bowel movement.tw
44. (bowel AND (function* OR habit* OR movement* OR symptom* OR motility OR stool*)).tw
45. colon transit.tw
46. intestinal motility.tw
47. OR/ 41-46
48. 40 AND 47
49. acupuncture/
50. exp acupuncture therapy/
51. electroacupuncture/
52. meridians/
53. acupuncture points/
54. acupunctur*.tw.
55. (electroacupuncture OR electro-acupuncture).tw.
56. acupoints.tw.
57. ((meridian OR non-meridian OR trigger) AND point*).tw.
58. OR/49-57
59. randomized controlled trial.pt
60. controlled clinical trial.pt
61. randomized.tw
62. placebo.tw
63. clinical trials as topic/
64. randomly .tw
65. trial.tw
66. OR/59-65
67. Animals/ NOT humans/
68. 65 NOT 67
69. 48 AND 58 AND 68

For peer review only

PRISMA-P 2015 checklist

Section and topic	Item No	Page	Checklist item
Administrative information			
Title			
Identification	1a	Page 1	Acupuncture for constipation in stroke patients: protocol of a systematic review and meta-analysis
Update	1b		No
Registration	2	Page 2	This protocol has been registered on PROSPERO (CRD42017076880).
Authors:			
Contact	3a	Page 1	Jingbo Zhai[1], Wei Mu[2], Jinhua Si[3], Yan Li[4], Chen Zhao[5], Hongcai Shang[6], Huanan Li[7,8]*, Guihua Tian[6*]<br>[1]Research institute of Traditional Chinese Medicine, Tianjin University of Traditional Chinese Medicine, 312 Anshanxi Road, Nankai District, Tianjin 300193, China;<br>[2]Department of Clinical Pharmacology, The Second Affiliated Hospital of Tianjin University of Traditional Chinese Medicine, 69 Zengchan Road, Hebei District, Tianjin 300250, China;<br>[3]Library of Tianjin University of Traditional Chinese Medicine, 312 Anshanxi Road, Nankai District, Tianjin 300193, China;<br>[4]School of Nursing, Tianjin University of Traditional Chinese Medicine, 312 Anshanxi Road, Nankai District, Tianjin 300193, China;<br>[5]Hong Kong Chinese Medicine Clinical study Centre, Hong Kong Baptist University, 7 Baptist

For peer review only

			University Road, Kowloon Tong, Hong Kong, China;<br>[6]Key Laboratory of Chinese Internal Medicine of Ministry of Education and Beijing, Dongzhimen Hospital, Beijing University of Chinese Medicine, Beijing 100700, China;<br>[7]First Teaching Hospital of Tianjin University of Traditional Chinese Medicine, 314 Anshanxi Road, Nankai District, Tianjin 300193, China;<br>[8]Laboratory for Biological Effects of Tuina, State Administration of Traditional Chinese Medicine, 314 Anshanxi Road, Nankai District, Tianjin 300193, China;<br>Correspondence to Huanan Li, lihuanan1984@126.com; Guihua Tian, Rosetgh@163.com
Contributions	3b	Page 8	JZ, HL and GT conceived the study. JZ, HL and GT provided general guidance to the drafting of the protocol. JZ and WM drafted the protocol. JS designed the search strategy. JZ, YL, CZ and HS drafted the manuscript. JZ, WM, JS, YL, CZ, HL, GT and HS reviewed and revised the manuscript. All authors have read and approved the final version of the manuscript.
Amendments	4	Page 9	We will provide the date of any amendment, a description of the change and the rationale in the event of protocol amendments.
Support:			
Sources	5a	Page 9	This study is supported by the National Natural Science Foundation of China [grant number 81703936] and Beijing Nova Program [grant number xx2014B049].
Sponsor	5b	Page 8	JZ, HL and GT are the sponsors.
Role of sponsor or funder	5c	Page 8	JZ, HL and GT conceived the study. JZ, HL and GT provided general guidance to the drafting of the protocol.
Introduction			

For peer review only

Rationale	6	Page 2-3	Stroke is one of the leading causes of death and disability worldwide. Constipation is one of the most common complications in patients with stroke. Constipation has a negative impact on the patient's psycholigical well-being and physical health. Conventional treatments may be associated with unwanted side effects, such as bloating, dehydration, a high recurrence rate after ceasing drugs. Many constipation patients seek help from alternative therapies.<br>Acupuncture has gained increased popularity for the management of constipation in Western countries. Overall, there is a lack of supportive evidence on the efficacy and safety of acupuncture for constipation in patients with stroke.
Objectives	7	Page 3	The aim of this study is to systematically review current available literature to assess the efficacy and safety of the acupuncture treatment for post-stroke constipation.
Methods			
Eligibility criteria	8	Page 3-5	We will only include randomized controlled trials (RCTs) which are more likely to provide unbiased information than other study designs. We will exclude quasi-randomized RCTs, such as those allocating by alphabetical order, alternate days of the week, or date of birth. Cross-over trials will be excluded because of potential for a carry-over effect. There is no restriction on language or publicaiton status.<br>We will include adults (over 18 years old) suffering from constipation after a first or recurrent stroke. Trials will be excluded in which patients have a prior history of constipation before the stroke diagnosis. Stroke is defined as 'rapidly developed signs of focal or global disturbance of cerebral funcation, lasting more than 24 hours or leading to death with no apparent cause other than that of vascular origin' according to WHO criteria. We will include stroke patients irrespective of any type (ischaemic or haemorrhagic) or phase (acute, subacute or chronic). Acute and subacute stroke is defined as less than six months since onset, and chronic stroke lasts more than six months since onset.<br>All of patients should be diagnosed as constipation according to at least one of the current or past definitions or guidelines of constipation, such as Rome □/□ diagnostic criteria or guidelines for clinical research on Chinese new herbal medicine. There is no restriction on age, sex or ethnicity of

For peer review only

			the enrolled subjects.<br>We will include trials using either traditional or contemporary acupuncture. Traditional acupuncture refers to needles inserted in classical meridian points. Contemporary acupuncture refers to needles inserted in non-meridian or trigger points regardless of the source of stimulation (for example, hand, electrical stimulation or fine needle) . We will exclude trials in which treatment without needling, such as acupressure, tap-pricking, point injection, laser acupuncture. No restrictions are imposed on times of treatment and length of treatment period.<br>The control interventions could be placebo acupuncture, sham acupuncture, no treatment, another active treatment or medication. Placebo acupuncture refers to a needle attached to the skin surface without penetrating the skin. Sham acupuncture is defined as a needle placed in an area close to but not in acupuncture points or subliminal skin electrostimulation via electrodes attached to the skin. We consider another active treatment or medication to be pharmacologic and nonpharmacoligic treatment or medication, such as laxatives, emollients, kubricants, lifestyle or dietary modification.
Information sources	9	Page 5	The published literature will be identified by searching Pubmed, Embase, Cochrane Central Register of Controlled Trials, and Web of science. CNKI, CBM, WANFANG, and VIP database will also be systematically searched to identify any relevant study published in China.<br>We will check the reference lists of identified relevant RCTs and reviewed articles for additional studies. We will contact experts in the field of stroke and constipation to identify any additional trials. The World Health Organization International Clincial trials Registry platform (ICTRP), ClinicalTrials.gov will also be checked to identifiy planned, ongoing or unpublished trials. Google Scholar will be searched to identify any grey literature.
Search strategy	10	Page 5	The search strategy is developed by a medical librarian (JS) according to key terms from previous literature reviews. The complete search strategy for the pubmed database is attached (appendix 1). The terms will be modified as necessary for other databases. We will not apply any language or date restrictions.

For peer review only

Study records			
Data management	11a	Page 6	Duplicates will be omitted using EndNote software (version X7.0). EndNote software will also be used to manage records. We will illustrate the selection process in a PRISMA diagram.
Selection process	11b	Page 6	Two review authors (WM and JS) will independently assess abstracts and titles of studies identified by literature search. Revelant studies will be selected against the predefined inclusion criteria. If necessary, reviewers will examine full-text reports to identify eligible studies. Any disagreement will be resolved by consensus.
Data collection process	11c	Page 6	Two review authors (YL and CZ) will independently extract data from the included studies. Calibration exercises will be conducted to ensure consistency across reviewers before starting the review. Where necessary we will contact study authors for clarification of data and additional information. Any disagreement will be resolved by consensus or consultation with a third review author.
Data items	12	Page 6	The following information will be extracted using a predetermined data form: general information (title, authors, country of study, funding, year of publication, registry number); details of study (aim, design, inclusion and exclusion criteria, method of randomization and allocation); study population (age, sex, sample size, number for analysis, type of stroke); intervention characteristics (type, duration, dose, follow-up time points, compliance); outcome (primary and secondary outcomes, time points, method of outcome assessments, blinding of outcome assessment).
Outcomes and prioritization	13	Page 5	The primary outcome measure will be frequency of bowel movement. Bowel movement frequency is defined as the mean number of spontaneous bowel movements per week.<br>Secondary outcome measures include proportion of patients with stool consistency, proportion of patients using rescue medication such as laxatives or rectal evacuants, Quality of life (QoL), and mean transit time.<br>Stool consistency will be defined by trialists or measured based on scales such as Bristol Stool Form Scale (BSFS).<br>QoL will be measured by generic or condition-specific scales, such as Short Form 36 Health

For peer review only

			Surveys (SF-36).<br>Transit time is defined as the time from the first perception of wanting to defaecate to the finish of defaecation.<br>We will sum up the number of adverse events (AEs), and calculate the proportion of AEs.
Risk of bias in individual studies	14	Page 6	Two authors (HL and HS) will independently assess the risk of bias using the approach recommended by Cochrane Handbook for Systematic Reviews of Interventions. The following risk of bias domains will be assessed: sequence generation (selection bias); allocation concealment (selection bias); blinding of participants and personnel (performance bias); blinding of outcome assessment (detection bias); incomplete outcome data (attrition bias); selective outcome reporting (reporting bias); other bias.<br>We will attempt to describe what is reported to have happened in each study for each domain of risk of bias. Thus, we will be able to provide the rationale for the judgement of whether this domain is at low, high, or unclear risk of bias. Where necessary we will contact authors of included studies for missing information or clarification.<br>If all domians are at low risk of bias, the overall risk of bias of individual studies will be categorised as low risk of bias. Otherwise, overall risk of bias will categorised as high risk of bias. The ‘Risk of bias’ summary will be presented graphically.
Data synthesis	15a	Page 7	We will combine more than one trial to estimate pooled intervention effect using the meta-analysis when studies examine the same intervention and outcomes with comparable methods in similar populations.
	15b	Page 7	We will pool the continuous data using the inverse variance method, and dichotomous data using the Mantel-Haenszel method.<br>We will use the fixed-effect model to combine data when statistical heterogeneity is low ($I^2 < 50\%$). However, when $I^2 > 50\%$, the random-effect model will be used to provide a more conservative estimate of effect.<br>All analyses will be conducted with Review Manager 5.3 software.

For peer review only

	15c	Page 8	We will perform the following subgroup analyses to investigate heterogeneity when sufficient data are available. We will conduct subgroup analyses based on age, sex, type of stroke (hemorrhagic and ischemic stroke), different definitions of constipation, phase of stroke (acute, subacute or chronic), and type of control group (placebo, sham acupuncture, no treatment or another active treament or medication).<br>The intervention effect will be analyzed using the Chi^2 test, with $P < 0.05$ indicating statistically significant differences between subgroups.<br>We will carry out sensitivity analyses to evaluate the robustness of the pooled results excluding trials with high risk of bias, and the option of using missing data (worst-case scenario analysis, extreme worst-case analysis, or extreme best-case analysis).
	15d	Page 7	If a meta-analysis is not possible, we will provide a narrative summary of the results from individual studies.
Meta-bias(es)	16	Page 7	When a meta-analysis includes 10 or more RCTs, we will assess asymmetry using funnel plots visually. In addtition, we will test asymmetry using the Harbord modified test for dichotomous outcomes and Egger test for continuous outcomes.
Confidence in cumulative evidence	17	Page 8	We will prepare 'Summary of findings' tables including a grade of the overall quality of the body of evidence for each outcome using GRADEpro GDT.<br>Two review authors will independently assess the quality of the body of evidence according to five GRADE criteria: study limitations, imprecision, inconsistency, indirectness, and publication bias. It will fall into one of four possible ratings (high, moderate, low and very low). Any discrepancies will be resolved by consensus or consultation with a third review author.

For peer review only

BMJ Open

Acupuncture for constipation in stroke patients: protocol of a systematic review and meta-analysis

Journal:	*BMJ Open*
Manuscript ID	bmjopen-2017-020400.R1
Article Type:	Protocol
Date Submitted by the Author:	23-Jan-2018

Complete List of Authors:	Zhai, Jingbo; Tianjin University of Traditional Chinese Medicine, Research institute of Traditional Chinese Medicine<br>Mu, Wei; The Second Affiliated Hospital of Tianjin University of Traditional Chinese Medicine, Department of Clinical Pharmacology<br>Si, Jinhua; Tianjin University of Traditional Chinese Medicine, Library<br>Li, Yan; Tianjin University of Traditional Chinese Medicine, School of Nursing<br>zhao, chen; Hong Kong Baptist University, Hong Kong Chinese Medicine Clinical study Centre<br>Shang, Hongcai; Dongzhimen Hospital, Beijing University of Chinese Medicine, Key Laboratory of Chinese Internal Medicine of Ministry of Education and Beijing<br>Li, Huanan; First Teaching Hospital of Tianjin University of Traditional Chinese Medicine, Laboratory for Biological Effects of Tuina<br>Tian, Guihua; Dongzhimen Hospital, Beijing University of Chinese Medicine, Key Laboratory of Chinese Internal Medicine of Ministry of Education and Beijing

**Primary Subject Heading**:	Cardiovascular medicine
Secondary Subject Heading:	Cardiovascular medicine, Complementary medicine
Keywords:	Stroke medicine < INTERNAL MEDICINE, constipation, Acupuncture, systematic review, meta-analysis

SCHOLARONE™ Manuscripts

Acupuncture for constipation in stroke patients: protocol of a systematic review and meta-analysis

Jingbo Zhai[1], Wei Mu[2], Jinhua Si[3], Yan Li[4], Chen Zhao[5], Hongcai Shang[6], Huanan Li[7,8]*, Guihua Tian[6*]

[1]Research institute of Traditional Chinese Medicine, Tianjin University of Traditional Chinese Medicine, 312 Anshanxi Road, Nankai District, Tianjin 300193, China;

[2]Department of Clinical Pharmacology, The Second Affiliated Hospital of Tianjin University of Traditional Chinese Medicine, 69 Zengchan Road, Hebei District, Tianjin 300250, China;

[3]Library of Tianjin University of Traditional Chinese Medicine, 312 Anshanxi Road, Nankai District, Tianjin 300193, China;

[4]School of Nursing, Tianjin University of Traditional Chinese Medicine, 312 Anshanxi Road, Nankai District, Tianjin 300193, China;

[5]Hong Kong Chinese Medicine Clinical study Centre, Hong Kong Baptist University, 7 Baptist University Road, Kowloon Tong, Hong Kong, China;

[6]Key Laboratory of Chinese Internal Medicine of Ministry of Education and Beijing, Dongzhimen Hospital, Beijing University of Chinese Medicine, Beijing 100700, China;

[7]First Teaching Hospital of Tianjin University of Traditional Chinese Medicine, 314 Anshanxi Road, Nankai District, Tianjin 300193, China;

[8]Laboratory for Biological Effects of Tuina, State Administration of Traditional Chinese Medicine, 314 Anshanxi Road, Nankai District, Tianjin 300193, China;

Correspondence to Huanan Li, lihuanan1984@126.com; Guihua Tian, Rosetgh@163.com

ABSTRACT

Introduction:

Constipation is one of the most common complications in patients with stroke. Acupuncture has gained increased popularity for the management of constipation. However, there is a lack of supportive evidence on the efficacy of acupuncture for post-stroke constipation. This systematic review aims to collect and critically appraise all the available evidence about the efficacy and safety of the acupuncture for

For peer review only

For peer review only - http://bmjopen.bmj.com/site/about/guidelines.xhtml

constipation in post-stroke patients.

Methods and analysis:

A comprehensive search of Pubmed, Embase, Cochrane Central Register of Controlled Trials, Web of science, four Chinese databases (CNKI, CBM, WANFANG, and VIP database), one Japanese medical database (CiNii) and one Korean medical database (OASIS) will be conducted to identify randomized controlled trials of acupuncture for constipation in post-stroke patients. There is no restriction on language or publication status.

The primary outcome measure will be frequency of bowel movement. The risk of bias will be assessed using the approach recommended by Cochrane Handbook for Systematic Reviews of Interventions.

We will conduct the meta-analysis to synthesise the evidence for each outcome, if possible. The heterogeneity will be statistically assessed using a Chi^2 test and I^2 statistic.

This protocol is developed following the guideline of PRISMA-P 2015.

Ethics and dissemination:

The ethical approval is not required because no primary data is collected. The findings will be presented at scientific conferences or a peer-reviewed scientific journal.

This protocol has been registered on PROSPERO (CRD42017076880).

Strengths and limitations of this study

- This review will provide a comprehensive assessment regarding the effect of acupuncture for constipation in patients with stroke.
- Only randomized controlled trials will be included which are more likely to provide unbiased information than other study designs.
- The reliability of the results will largely depend on the comprehensiveness and the methodological quality of the primary studies included in this review.

Introduction

Stroke is one of the leading causes of death and disability worldwide[1]. The incidence of stroke is subject to large variation globally[2]. The overall incidence in low to middle income countries exceeded that in high-income countries by 20% from 2000 to 2008[2].

Constipation is one of the most common complications in patients with stroke[3]. The evidence from a previous systematic review shows that the incidence of constipation in stroke patients is 48% (95% confidence interval, 33% to 63%)[4]. The incidence of constipation in patients with hemorrhagic stroke is higher than that in patients with ischemic type[4]. The incidence in rehabilitation stage is higher than that in acute stage[4].

Constipation has a negative impact on the patient’s psychological well-being and physical health, restricts social activities, reduces quality of life, contributes to a poor

For peer review only - http://bmjopen.bmj.com/site/about/guidelines.xhtml

outcome and is associated with high healthcare costs[4-6].

Stool softeners, prokinetic agents, osmotic and stimulant laxatives, and lifestyle or dietary modification are common treatments for constipation[7]. Conventional treatments may be associated with unwanted side effects, such as bloating, dehydration, a high recurrence rate after ceasing drugs[7,8]. Most of patients with chronic constipation are not satisfied with current treatment options in Europe[9]. Many constipation patients seek help from alternative therapies[9].

Acupuncture, as an important part of complementary and alternative medicine (CAM), has gained increased popularity for the management of constipation in Western countries[9]. There are some possible mechanisms of acupuncture for constipation[8]. Acupuncture can promote intestinal canal peristalsis through contacting the intestinal wall, and regulating nervous and body fluids[8]. Acupuncture can increase rectal internal pressure to restore the defecation sense by stimulating parasympathetic nerve[8].

A systematic review shows that acupuncture is safe for chronic functional constipation[10]. A randomized trial supports the use of electroacupuncture for chronic severe functional constipation[11]. However, whether the evidence is transferrable to the stroke population remains unclear.

The recent studies mainly focus on the incidence of constipation after stroke[4,5]. The management strategies for constipation in stroke patients remains poorly investigated[3].

A 2014 systematic review evaluated the efficacy and safety of acupuncture and moxibustion for post-stroke constipation[12]. This review had evident flaws that threatened the authenticity of their findings. First, meta-analysis found that acupuncture and moxibustion were significantly more effective than other treatments (OR=2.10, 95% credible interval 1.25 to 3.54, P=0.005) for constipation in stroke patients. Acupuncture and moxibustion were addressed as a whole, and the efficacy of acupuncture alone was not systematically investigated, despite that five of the eight included trials compared acupuncture alone with another treatment. Second, the methodological quality of eight included articles was very poor. Third, the total effective rate, a subjective outcome measure, was chosed as the primary outcome. Forth, the sample size was small. To our knowledge, several new RCTs have been published since the meta-analysis was published[13,14]. Overall, there is a lack of supportive evidence on the efficacy and safety of acupuncture for constipation in patients with stroke.

The aim of this study is to systematically review current available literature to assess the efficacy and safety of the acupuncture treatment for post-stroke constipation.

Methods:

This protocol is developed following the guideline of PRISMA-P 2015[15].

Inclusion criteria

Types of studies

For peer review only - http://bmjopen.bmj.com/site/about/guidelines.xhtml

We will only include randomized controlled trials (RCTs) which are more likely to provide unbiased information than other study designs[16]. We will exclude quasi-randomized RCTs, such as those allocating by alphabetical order, alternate days of the week, or date of birth. Cross-over trials will be excluded because of potential for a carry-over effect. There is no restriction on language or publication status.

Types of participants

We will include adults (over 18 years old) suffering from constipation after a first or recurrent stroke. We also consider RCTs in which a prior history of constipation before the stroke diagnosis is not investigated, but excluded trials reporting on patients with a prior history of constipation before the stroke diagnosis. Stroke is defined as 'rapidly developed signs of focal or global disturbance of cerebral function, lasting more than 24 hours or leading to death with no apparent cause other than that of vascular origin' according to WHO criteria[17]. We will include stroke patients irrespective of any type (ischemic or hemorrhagic) or phase (acute, subacute or chronic). Acute and subacute stroke is defined as less than six months since onset, and chronic stroke lasts more than six months since onset[18].

All of patients should be diagnosed as constipation according to at least one of the current or past definitions or guidelines of constipation, such as Rome □/□ diagnostic criteria or guidelines for clinical research on Chinese new herbal medicine[12].

There is no restriction on age, sex or ethnicity of the enrolled subjects.

Types of interventions

Experimental interventions

We will include trials using either traditional or contemporary acupuncture. Traditional acupuncture refers to needles inserted in classical meridian points[19]. Contemporary acupuncture refers to needles inserted in non-meridian or trigger points regardless of the source of stimulation (for example, hand, electrical stimulation or fine needle) [19]. We will exclude trials in which treatment without needling, such as acupressure, tap-pricking, point injection, laser acupuncture. No restrictions are imposed on times of treatment and length of treatment period.

Comparator interventions

The control interventions could be placebo acupuncture, sham acupuncture, no treatment, another active treatment or medication.

Placebo acupuncture refers to a needle attached to the skin surface without penetrating the skin[20].

Sham acupuncture is defined as a needle placed in an area close to but not in acupuncture points or subliminal skin electrostimulation via electrodes attached to the skin[20].

We consider another active treatment or medication to be pharmacologic and nonpharmacologic treatment or medication, such as laxatives, emollients, lubricants, lifestyle or dietary modification.

For peer review only

For peer review only - http://bmjopen.bmj.com/site/about/guidelines.xhtml

We will investigate the comparisons listed below:

1. acupuncture only compared with no treatment;
2. acupuncture only compared with placebo or sham treatment;
3. acupuncture plus another active treatment or medication compared with another active treatment or medication alone;
4. acupuncture plus another active treatment or medication compared with placebo or sham treatment plus another active treatment or medication.

Types of outcome measures:

Primary outcome

The primary outcome measure will be frequency of bowel movement[21,22]. Bowel movement frequency is defined as the mean number of spontaneous bowel movements per week[23].

Secondary outcome

Secondary outcome measures include proportion of patients with stool consistency, proportion of patients using rescue medication such as laxatives or rectal evacuants, Quality of life (QoL), and mean transit time.

Stool consistency will be defined by trialists or measured based on scales such as Bristol Stool Form Scale (BSFS) [24].

QoL will be measured by generic or condition-specific scales, such as Short Form 36 Health Surveys (SF-36)[25].

Transit time is defined as the time from the first perception of wanting to defecate to the finish of defecation[26].

We will sum up the number of adverse events (AEs), and calculate the proportion of AEs[23].

Search strategy

Electronic searches

The published literature will be identified by searching Pubmed, Embase, Cochrane Central Register of Controlled Trials, and Web of science. Four Chinese databases (CNKI, CBM, WANFANG, and VIP database), one Japanese medical database (CiNii) and one Korean medical database (OASIS) will also be systematically searched to identify any relevant study.

The search strategy is developed by a medical librarian (JS) according to key terms from previous literature reviews[27,28]. The detailed search strategy is attached (appendix 1). The terms will be modified as necessary for other databases. We will not apply any language or date restrictions.

Searching other resources

We will check the reference lists of identified relevant RCTs and reviews for additional studies. We will contact experts in the field of stroke and constipation to identify any additional trials.

The World Health Organization International Clinical trials Registry platform

For peer review only - http://bmjopen.bmj.com/site/about/guidelines.xhtml

(ICTRP), ClinicalTrials.gov will also be checked to identify planned, ongoing or unpublished trials. Google Scholar will be searched to identify any grey literature.

Data collection and analysis

Selection of studies

Two review authors (WM and JS) will independently assess abstracts and titles of studies identified by literature search. Duplicates will be omitted using EndNote software (version X7.0)[29]. Revelant studies will be selected against the predefined inclusion criteria. If necessary, reviewers will examine full-text reports to identify eligible studies. EndNote software will also be used to manage records. We will illustrate the selection process in a PRISMA diagram[30]. Any disagreement will be resolved by consensus.

Data extraction and management

Two review authors (YL and CZ) will independently extract data from the included studies. Calibration exercises will be conducted to ensure consistency across reviewers before starting the review. The following information will be extracted using a predetermined data form: general information (title, authors, country of study, funding, year of publication, registry number); details of study (aim, design, inclusion and exclusion criteria, method of randomization and allocation); study population (age, sex, sample size, number for analysis, type of stroke, phase of stroke); intervention characteristics (type, duration, dose, follow-up time points, compliance); outcome (primary and secondary outcomes, time points, method of outcome assessments, blinding of outcome assessment, adverse effects).

It is possible that patients in different phases of stroke were enrolled in one trial. Whenever necessary the authors of the original trial will be contacted for additional information and clarification of the data. Any disagreement will be resolved by consensus or consultation with a third review author.

Assessment of risk of bias in included studies

Two authors (HL and HS) will independently assess the risk of bias using the approach recommended by Cochrane Handbook for Systematic Reviews of Interventions[31]. The following risk of bias domains will be assessed: sequence generation (selection bias); allocation concealment (selection bias); blinding of participants and personnel (performance bias); blinding of outcome assessment (detection bias); incomplete outcome data (attrition bias); selective outcome reporting (reporting bias); other bias.

We will attempt to describe what is reported to have happened in each study for each domain of risk of bias. Thus, we will be able to provide the rationale for the judgement of whether this domain is at low, high, or unclear risk of bias. Where necessary we will contact authors of included studies for missing information or clarification.

If all domains are at low risk of bias, the overall risk of bias of individual studies will be categorised as low risk of bias. Otherwise, overall risk of bias will be

For peer review only - http://bmjopen.bmj.com/site/about/guidelines.xhtml

categorised as high risk of bias[32]. The 'Risk of bias' summary will be presented graphically.

Measures of treatment effect

We will use the risk ratio (RR) with 95% confidence interval (CI) to express the estimate of the effect for dichotomous outcome.

For continuous outcome, we will express the estimate of the effect as mean difference (MD) with 95% CI. When the same outcome is measured in a variety of ways, the standardized mean difference (SMD) with 95% CI will be used to express the size of the intervention effect.

Dealing with missing data

We will attempt to contact study authors for missing data or clarification, where feasible. The following strategies will be used to evaluate the potential influence of missing data[32].

1. Worst-case scenario analysis: all participants with missing data counted as failures.
2. Extreme worst-case analysis: participants with missing data in experimental group counted as failures and participants with missing data in control group counted as successes.
3. Extreme best-case analysis: participants with missing data in experimental group counted as successes and participants with missing data in control group counted as failures.

Assessment of heterogeneity

We will assess the statistical heterogeneity using a Chi^2 test[33]. In addition, we will quantify heterogeneity using the I^2 statistic value which ranges from 0% to 100%[34]. $P < 0.1$ of Chi^2 test or $I^2 > 50\%$ indicates statistically significant heterogeneity[33,34]. Potential clinical heterogeneity will be assessed by prespecified subgroup analyses.

Assessment of reporting biases

When a meta-analysis includes 10 or more RCTs, we will assess asymmetry using funnel plots visually[32]. In addition, we will test asymmetry using the Harbord modified test for dichotomous outcomes and Egger test for continuous outcomes[32].

Data synthesis

We will combine more than one trial to estimate pooled intervention effect using the meta-analysis when studies examine the same intervention and outcomes with comparable methods in similar populations[31].

We will pool the continuous data using the inverse variance method, and dichotomous data using the Mantel-Haenszel method[31].

We will use the fixed-effect model to combine data when statistical heterogeneity is low. However, when $P < 0.1$ or $I^2 > 50\%$, the random-effect model will be used to provide a more conservative estimate of effect[35].

For peer review only - http://bmjopen.bmj.com/site/about/guidelines.xhtml

All analyses will be conducted with Review Manager 5.3 software. If a meta-analysis is not possible, we will provide a narrative summary of the results from individual studies.

Subgroup analysis and investigation of heterogeneity

We will perform the following subgroup analyses to investigate heterogeneity when sufficient data are available. We will conduct subgroup analyses based on age, sex, type of stroke (hemorrhagic and ischemic stroke), different definitions of constipation, phase of stroke (acute, subacute or chronic), type of acupuncture (manual acupuncture, electroacupuncture, etc.), and type of control group (placebo, sham acupuncture, no treatment or another active treament or medication). A subgroup analysis based on population with different diet habbits is under plan considering that diet habits may play an important role in constipation development.

The intervention effect will be analyzed using the Chi^2 test, with $P < 0.05$ indicating statistically significant differences between subgroups.

Sensitivity analysis

We will perform sensitivity analyses to evaluate the robustness of the pooled results excluding trials with high risk of bias, and trials in which a prior history of constipation before the stroke diagnosis is not investigated, and the option of using missing data (worst-case scenario analysis, extreme worst-case analysis, or extreme best-case analysis) [32,34].

Summary of findings table

We will prepare 'Summary of findings' tables including a grade of the overall quality of the body of evidence for each outcome using GRADEpro GDT[36].

Two review authors will independently assess the quality of the body of evidence according to five GRADE criteria: study limitations, imprecision, inconsistency, indirectness, and publication bias. It will fall into one of four possible ratings (high, moderate, low and very low). Any discrepancy will be resolved by consensus or consultation with a third review author.

Amendments

We will provide the date of any amendment, a description of the change and the rationale in the event of protocol amendments.

Ethics and dissemination

Ethical approval is not required because no primary data is collected.

This review will provide a comprehensive assessment regarding the effect of acupuncture for constipation in patients with stroke. The results will be fundamental for reliable recommendations in the management of post-stroke constipation.

We will present findings from this systematic review at scientific conferences and publish the findings in a peer-reviewed scientific journal according to the PRISMA guidelines.

Protocol registration: This protocol has been registered on PROSPERO

For peer review only - http://bmjopen.bmj.com/site/about/guidelines.xhtml

(CRD42017076880).

Contributors:

JZ, HL and GT conceived the study. JZ, HL and GT provided general guidance to the drafting of the protocol. JZ and WM drafted the protocol. JS designed the search strategy. JZ, YL, CZ and HS drafted the manuscript. JZ, WM, JS, YL, CZ, HL, GT and HS reviewed and revised the manuscript. All authors have read and approved the final version of the manuscript.

Funding:

This study is supported by the National Natural Science Foundation of China [grant number 81373762], the National Natural Science Foundation of China [grant number 81603495], the National Natural Science Foundation of China [grant number 81703936] and Beijing Nova Program [grant number xx2014B049].

Competing interests: None declared.

Ethics approval: Not required.

Provenance and peer review: Not commissioned; externally peer reviewed.

Data sharing statement: We will publish findings from this systematic review in a peer-reviewed scientific journal, and data set will be made freely available.

Open access: This is an Open Access article distributed in accordance with the terms of the Creative Commons Attribution (CC BY 4.0) license, which permits others to distribute, remix, adapt and build upon this work, for commercial use, provided the original work is properly cited. See: http://creativecommons.org/licenses/by/4.0/

References:

[1] Kathleen Strong, Colin Mathers, Ruth Bonita. Preventing stroke: saving lives around the world. Lancet 2007;6:182-187.

[2] Valery L Feigin, Carlene M M Lawes, Derrick A Bennett, et al. Worldwide stroke incidence and early case fatality reported in 56 population-based studies: a systematic review. Lancet Neurol 2009;8(4):355-369.

[3] Su Fee Lim, Charmaine Childs. A systematic review of the effectiveness of bowel management strategies for constipation in adults with stroke. Int J Nurs Stud 2013;50(7):1004-1010.

[4] Jianxiang Li, Mengguo Yuan, Yunfang Liu, et al. Incidence of constipation in stroke patients: A systematic review and meta-analysis. Medicine 2017;96:e7225.

[5] Yongjing Su, Xiaoyan Zhang, Jinsheng Zeng, et al. New-Onset Constipation at Acute Stage After First Stroke Incidence, Risk Factors, and Impact on the Stroke Outcome. Stroke 2009;40(4):1304-1309.

For peer review only - http://bmjopen.bmj.com/site/about/guidelines.xhtml

[6] Umar Shariff, Omar M Aboumarzouk, Richard L Nelson. Antibiotics for the management of constipation predominant irritable bowel syndrome and chronic constipation. Cochrane Database Syst Rev 2009;(1):CD007518.

[7] Shengli Zhou, Xiulai Zhang, Jinghua Wang. Comparison of electroacupuncture and medical treatment for functional constipation: a systematic review and meta-analysis. Acupunct Med 2017;35(5):324-331.

[8] Chaoxian Zhang, Like Guo, Xiaofeng Guo, et al. Short and long-term efficacy of combining Fuzhengliqi mixture with acupuncture in treatment of functional constipation. J Tradit Chin Med 2013;33(1):51-59.

[9] Tack J, Mueller-Lissner S, Dubois D, Schenck F. Only 27% of European patients with chronic constipation are satisfied with current treatment options. Gut 2009; 58(Suppl □):A181.

[10] Tao Zhang, Tony Y. Chon, Baoyan Liu, et al. Efficacy of Acupuncture for Chronic Constipation: A Systematic Review. Am J Chin Med 2013;41(4):717-742.

[11] Zhishun Liu, Shiyan Yan, Jiani Wu, et al. Acupuncture for Chronic Severe Functional Constipation: A randomized trial. Ann Intern Med 2016;165(11):761-769.

[12] Yang Jipeng, Liu Jingying, Gu Hongyan, et al. Randomized controlled trials of acupuncture and moxibustion for post-stroke constipation: a meta analysis. Zhongguo Zhen Jiu 2014;34(8):833-836.

[13] Wang Chengwei, Liu Mengyue, Wen Qian, et al. Clinical observation of preventing and treating constipation by electroacupunture intervention in ischemic stroke patients at acute stage. Zhongguo Zhen Jiu 2015;35(5):430-434.

[14] Liu Haifei, Song Fengjun, Ye Bihong. Therapeutic observation of acupuncture at He-Sea and Front-Mu points for post-stroke constipation. Shanghai J Acu-mox 2016;35(2):160-161.

[15] Larissa Shamseer, David Moher, Mike Clarke, et al. Preferred reporting items for systematic review and meta-analysis protocols (PRISMA-P) 2015: elaboration and explanation. BMJ 2015;349:g7647.

[16] Lise Lotte Gluud, Marsha Y Morgan. Endoscopic therapy and beta-blockers for secondary prevention in adults with cirrhosis and oesophageal varices. Cochrane Database Syst Rev 2017;6:CD012694.

[17] Hatano S. Experience from a multicentre stroke register: a preliminary report. Bull World Health Organ 1976;54(5):541–553.

[18] Ryo Momosaki, Naoki Yamada, Erika Ota, et al. Repetitive peripheral magnetic stimulation for activities of daily living and functional ability in people after stroke. Cochrane Database Syst Rev 2017;6:CD011968.

[19] Cheong YC, Dix S, Hung Yu Ng E, et al. Acupuncture and assisted reproductive technology. Cochrane Database Syst Rev 2013;7:CD006920.

[20] Daniel KL Cheuk, Virginia Wong, Wen Xiong Chen. Acupuncture for autism spectrum disorders. Cochrane Database Syst Rev 2011;9:CD007849.

[21] Sue Woodward, Christine Norton, Pauline Chiarelli. Biofeedback for treatment of chronic idiopathic constipation in adults. Cochrane Database Syst Rev 2014;3:CD008486.

[22] Eunice B Turawa, Alfred Musekiwa, Anke C Rohwer. Interventions for

For peer review only

For peer review only - http://bmjopen.bmj.com/site/about/guidelines.xhtml

preventing postpartum constipation. Cochrane Database Syst Rev 2015;9:CD011625.
[23] Min Chen, Hui Zheng, Juan Li, et al. Non-pharmacological treatments for adult patients with functional constipation: a systematic review protocol. BMJ Open 2014;4(6):e004982.
[24] Larry E. Miller, Alvin lbarra, Arthur C. Ouwehand, et al. Normative values for stool frequency and form using Rome III diagnostic criteria for functional constipation in adults: systematic review with meta-analysis. Ann Gastroenterol 2017;30(2):161-167.
[25] Maureen Coggrave, Christine Norton, June D Cody. Management of faecal incontinence and constipation in adults with central neurological diseases. Cochrane Database Syst Rev 2014;1:CD002115.
[26] Qianhua Zheng, Hui Zheng, Lingyun Lu, et al. Acupuncture for functional constipation: protocol of an individual patient data meta-analysis. BMJ Open 2015;5(5):e007137.
[27] Zhang SK, Liu M, Asplund K, et al. Acupuncture for acute stroke. Cochrane Database Syst Rev 2005;2:CD003317.
[28] Omar M Aboumarzouk, Trisha Agarwal, Ramez Antakia, et al. Cisapride for Intestinal Constipation. Cochrane Database Syst Rev 2011;1:CD007780.
[29] Guomin Song, Xiaoling Liu, Wei Bian, et al. Systematic review with network meta-analysis: comparative efficacy of different enteral immunonutrition formulas in patients underwent gastrectomy. Oncotarget 2017;8(14):23376-23388.
[30] Alessandro Liberati, Douglas G. Altman, Jennifer Tetzlaff, et al. The PRISMA Statement for Reporting Systematic Reviews and Meta-Analyses of Studies That Evaluate Health Care Interventions: Explanation and Elaboration. Plos Med 2009; 6(7):e1000100.
[31] Higgins JPT, Green S, eds. Cochrane handbook for systematic reviews of interventions version 5.1.0. [updated March 2011]. The Cochrane Collaboration, 2011. http://www.handbook.cochrane.org.
[32] Luise Aamann, Gitte Dam, Anders Rinnov, et al. Physical exercise for people with cirrhosis. Cochrane Database Syst Rev 2017;6:CD012678.
[33] Robert P Anthonappa, Paul F Ashley, Debbie L Bonetti, et al. Non-pharmacological interventions for managing dental anxiety in children. Cochrane Database Syst Rev2017;6:CD012676.
[34] Boonstra A, van Dulmen-den Broeder E, Rovers MM, et al. Severe fatigue in childhood cancer survivors. Cochrane Database Syst Rev 2017;6:CD012681.
[35] Browning GG, Howell P, Whitmer WM, et al. Unilateral versus bilateral hearing aids for bilateral hearing impairment in adults. Cochrane Database Syst Rev 2017;5:CD012665.
[36] GRADEpro GDT. GRADEpro Guideline Development Tool. 2015. https://gradepro.org.

For peer review only

For peer review only - http://bmjopen.bmj.com/site/about/guidelines.xhtml

Appendix 1.
Pubmed Search strategy:

1. Cerebrovascular Disorders/
2. Exp Basal Ganglia Cerebrovascular Disease/
3. Exp Brain Ischemia/
4. Exp Carotid Artery Diseases/
5. Exp Cerebrovascular Trauma/
6. Exp Intracranial Arteriovenous Malformations/
7. Exp Intracranial Arterial Diseases/
8. Exp Intracranial Embolism and Thrombosis/
9. Exp Intracranial Hemorrhages
10. Stroke/
11. Exp Brain Infarction/
12. Stroke, Lacunar/
13. Vasospasm, Intracranial
14. Vertebral Artery Dissection/
15. Exp Hypoxia, Brain/
16. (stroke* OR post stroke OR poststroke OR post-stroke OR apoplex* OR cerebrovasc* OR CVA OR SAH OR cerebral vasc*).tw
17. ((brain OR cerebr* OR cerebell* OR vertebrobasil* OR hemispher* OR intracran* OR intracerebral OR infratentorial OR supratentorial OR middle cerebr* OR mca* OR anterior circulation OR basilar artery OR vertebral artery) AND (Ischemi* OR infarct*OR thrombos*OR thromboem* OR emboli*OR occlus* OR hypoxi*)).tw
18. ((Brain* OR cerebr* OR cerebell* OR intracerebral OR intracran* OR parenchymal OR intraparenchymal OR intraventricular OR infratentorial OR supratentorial OR basal gangli* OR putaminal OR putamen OR posterior fossa OR hemisphere* OR subarachnoid)) AND (haemorrhag* OR hemorrhag* OR haematoma* OR hematoma* OR bleed*)) .tw
19. Exp Hemiplegia/
20. Exp Paresis/
21. Exp Aphasia/
22. Exp Gait Disorders, Neurologic/
23. (Hemipar* OR hemipleg* OR paresis OR paretic OR aphasi* OR dysphasi*).tw
24. Exp Brain Damage, Chronic"/
25. Brain Injuries/
26. Exp Brain Concussion/
27. Exp Brain Hemorrhage,Traumatic/
28. Brain Injury, Chronic/
29. Diffuse Axonal Injury/
30. Craniocerebral Trauma/
31. Exp Head Injuries, Closed/
32. Exp Intracranial Hemorrhage, Traumatic/
33. Exp Brain Abscess/

For peer review only

34. Exp Central Nervous System Infections/
35. Exp Encephalitis/
36. Exp Meningitis/
37. (encephalitis OR meningitis OR head injur*).tw
38. Exp Brain Neoplasms/
39. ((brain OR cerebr*) AND (injur* OR hypoxi* OR damage* OR concussion OR trauma* OR neoplasm* OR lesion* OR tumor* OR tumour* OR cancer* OR infection)).tw
40. OR/ 1- 39
41. Constipation/
42. (Impaction OR obstipation OR costiveness OR defecation OR evacuation).tw
43. delayed bowel movement.tw
44. (bowel AND (function* OR habit* OR movement* OR symptom* OR motility OR stool*)).tw
45. colon transit.tw
46. intestinal motility.tw
47. OR/ 41-46
48. 40 AND 47
49. acupuncture/
50. exp acupuncture therapy/
51. electroacupuncture/
52. meridians/
53. acupuncture points/
54. acupunctur*.tw.
55. (electroacupuncture OR electro-acupuncture).tw.
56. acupoints.tw.
57. ((meridian OR non-meridian OR trigger) AND point*).tw.
58. OR/49-57
59. randomized controlled trial.pt
60. controlled clinical trial.pt
61. randomized.tw
62. placebo.tw
63. clinical trials as topic/
64. randomly .tw
65. trial.tw
66. OR/59-65
67. Animals/ NOT humans/
68. 65 NOT 67
69. 48 AND 58 AND 68

CBM Search strategy:
#1 中风 OR 卒中 OR 脑*塞 OR 脑*血 OR 脑*栓 OR 蛛网膜下腔出血
#2 主题词=中风/全部副主题词
#3 主题词=卒中/全部副主题词

For peer review only

#4 主题词=梗塞，大脑前动脉/全部副主题词
#5 主题词=梗塞，大脑中动脉/全部副主题词
#6 主题词=梗塞，大脑后动脉/全部副主题词
#7 主题词=蛛网膜下腔出血/全部副主题词
#8 #1~#7/OR
#9 随机 OR 盲法 OR 安慰剂
#10 主题词=随机对照试验[文献类型]
#11 主题词=随机分配
#12 主题词=随机对照试验/全部副主题词
#13 #9~#12/OR
#14 针刺 OR 电针 OR 火针 OR 头针 OR 毫针 OR 手捻针 OR 芒针 OR 巨针 OR 体针 OR 温针 OR 针灸
#15 主题词=针刺/全部副主题词
#16 主题词=针刺穴位/全部副主题词
#17 主题词=针刺疗法/全部副主题词
#18 主题词=针灸疗法/全部副主题词
#19 #14~#18/OR
#20 便秘 OR 排便 OR 腹胀 OR 腹痛
#21 主题词=便秘/全部副主题词
#22 主题词=排便/全部副主题词
#23 #20~#22/OR
#24 #8 AND #13 AND #23

CNKI Search strategy:
(SU=中风 OR SU=卒中 OR SU=脑梗 OR SU=脑栓塞 OR SU=脑出血 OR SU=脑血栓 OR SU=蛛网膜下腔出血) AND (SU=针刺 OR SU=电针 OR SU=火针 OR SU=头针 OR SU=毫针 OR SU=手捻针 OR SU=芒针 OR SU=巨针 OR SU=体针 OR SU=温针 OR SU=针灸) AND (SU=便秘 OR SU=排便 OR SU=腹胀 OR SU=腹痛) AND (SU=随机 OR FT=随机)

Wanfang Search strategy:
主题:(中风+卒中+脑梗+脑栓塞+脑出血+脑血栓+蛛网膜下腔出血)*主题:(针刺+电针+火针+头针+毫针+手捻针+芒针+巨针+体针+温针+针灸)*主题:(便秘+排便+腹胀+腹痛)*随机

Vip Search strategy:
M=(中风+卒中+脑梗+脑栓塞+脑出血+脑血栓+蛛网膜下腔出血)*M=(针刺+电针+火针+头针+毫针+手捻针+芒针+巨针+体针+温针+针灸)*M=(便秘+排便+腹胀+腹痛)*U=随机

For peer review only

PRISMA-P 2015 checklist

Section and topic	Item No	Page	Checklist item
Administrative information			
Title			
Identification	1a	Page 1	Acupuncture for constipation in stroke patients: protocol of a systematic review and meta-analysis
Update	1b		No
Registration	2	Page 2	This protocol has been registered on PROSPERO (CRD42017076880).
Authors:			
Contact	3a	Page 1	Jingbo Zhai[1], Wei Mu[2], Jinhua Si[3], Yan Li[4], Chen Zhao[5], Hongcai Shang[6], Huanan Li[7,8]*, Guihua Tian[6*]<br>[1]Research institute of Traditional Chinese Medicine, Tianjin University of Traditional Chinese Medicine, 312 Anshanxi Road, Nankai District, Tianjin 300193, China;<br>[2]Department of Clinical Pharmacology, The Second Affiliated Hospital of Tianjin University of Traditional Chinese Medicine, 69 Zengchan Road, Hebei District, Tianjin 300250, China;<br>[3]Library of Tianjin University of Traditional Chinese Medicine, 312 Anshanxi Road, Nankai District, Tianjin 300193, China;<br>[4]School of Nursing, Tianjin University of Traditional Chinese Medicine, 312 Anshanxi Road, Nankai District, Tianjin 300193, China;<br>[5]Hong Kong Chinese Medicine Clinical study Centre, Hong Kong Baptist University, 7 Baptist

For peer review only

			University Road, Kowloon Tong, Hong Kong, China;<br>[6]Key Laboratory of Chinese Internal Medicine of Ministry of Education and Beijing, Dongzhimen Hospital, Beijing University of Chinese Medicine, Beijing 100700, China;<br>[7]First Teaching Hospital of Tianjin University of Traditional Chinese Medicine, 314 Anshanxi Road, Nankai District, Tianjin 300193, China;<br>[8]Laboratory for Biological Effects of Tuina, State Administration of Traditional Chinese Medicine, 314 Anshanxi Road, Nankai District, Tianjin 300193, China;<br>Correspondence to Huanan Li, lihuanan1984@126.com; Guihua Tian, Rosetgh@163.com
Contributions	3b	Page 9	JZ, HL and GT conceived the study. JZ, HL and GT provided general guidance to the drafting of the protocol. JZ and WM drafted the protocol. JS designed the search strategy. JZ, YL, CZ and HS drafted the manuscript. JZ, WM, JS, YL, CZ, HL, GT and HS reviewed and revised the manuscript. All authors have read and approved the final version of the manuscript.
Amendments	4	Page 8	We will provide the date of any amendment, a description of the change and the rationale in the event of protocol amendments.
Support:			
Sources	5a	Page 9	This study is supported by the National Natural Science Foundation of China [grant number 81373762], the National Natural Science Foundation of China [grant number 81603495], the National Natural Science Foundation of China [grant number 81703936] and Beijing Nova Program [grant number xx2014B049].
Sponsor	5b	Page 9	JZ, HL and GT are the sponsors.
Role of sponsor or	5c	Page 9	JZ, HL and GT conceived the study. JZ, HL and GT provided general guidance to the drafting of the protocol.

For peer review only

funder			
Introduction			
Rationale	6	Page 2-3	Stroke is one of the leading causes of death and disability worldwide. Constipation is one of the most common complications in patients with stroke. Constipation has a negative impact on the patient's psycholigical well-being and physical health. Conventional treatments may be associated with unwanted side effects, such as bloating, dehydration, a high recurrence rate after ceasing drugs. Many constipation patients seek help from alternative therapies.<br>Acupuncture has gained increased popularity for the management of constipation in Western countries. Overall, there is a lack of supportive evidence on the efficacy and safety of acupuncture for constipation in patients with stroke.
Objectives	7	Page 3	The aim of this study is to systematically review current available literature to assess the efficacy and safety of the acupuncture treatment for post-stroke constipation.
Methods			
Eligibility criteria	8	Page 3-5	We will only include randomized controlled trials (RCTs) which are more likely to provide unbiased information than other study designs. We will exclude quasi-randomized RCTs, such as those allocating by alphabetical order, alternate days of the week, or date of birth. Cross-over trials will be excluded because of potential for a carry-over effect. There is no restriction on language or publicaiton status.<br>We will include adults (over 18 years old) suffering from constipation after a first or recurrent stroke. We also consider RCTs in which a prior history of constipation before the stroke diagnosis is not investigated, but excluded trials reporting on patients with a prior history of constipation before the stroke diagnosis. Stroke is defined as 'rapidly developed signs of focal or global disturbance of cerebral funcation, lasting more than 24 hours or leading to death with no apparent cause other than that of vascular origin' according to WHO criteria. We will include stroke patients irrespective of any type (ischaemic or haemorrhagic) or phase (acute, subacute or chronic). Acute and subacute stroke is defined as less than six months since onset, and chronic stroke lasts more than six months since onset.

For peer review only

			All of patients should be diagnosed as constipation according to at least one of the current or past definitions or guidelines of constipation, such as Rome □/□ diagnostic criteria or guidelines for clinical research on Chinese new herbal medicine. There is no restriction on age, sex or ethnicity of the enrolled subjects.<br>We will include trials using either traditional or contemporary acupuncture. Traditional acupuncture refers to needles inserted in classical meridian points. Contemporary acupuncture refers to needles inserted in non-meridian or trigger points regardless of the source of stimulation (for example, hand, electrical stimulation or fine needle) . We will exclude trials in which treatment without needling, such as acupressure, tap-pricking, point injection, laser acupuncture. No restrictions are imposed on times of treatment and length of treatment period.<br>The control interventions could be placebo acupuncture, sham acupuncture, no treatment, another active treatment or medication. Placebo acupuncture refers to a needle attached to the skin surface without penetrating the skin. Sham acupuncture is defined as a needle placed in an area close to but not in acupuncture points or subliminal skin electrostimulation via electrodes attached to the skin. We consider another active treatment or medication to be pharmacologic and nonpharmacoligic treatment or medication, such as laxatives, emollients, kubricants, lifestyle or dietary modification.
Information sources	9	Page 5	The published literature will be identified by searching Pubmed, Embase, Cochrane Central Register of Controlled Trials, and Web of science. Four Chinese databases (CNKI, CBM, WANFANG, and VIP database), one Japanese medical database (CiNii) and one Korean medical database (OASIS) will also be systematically searched to identify any relevant study.<br>We will check the reference lists of identified relevant RCTs and reviewed articles for additional studies. We will contact experts in the field of stroke and constipation to identify any additional trials. The World Health Organization International Clincial trials Registry platform (ICTRP), ClinicalTrials.gov will also be checked to identifiy planned, ongoing or unpublished trials. Google Scholar will be searched to identify any grey literature.

For peer review only

Search strategy	10	Page 5	The search strategy is developed by a medical librarian (JS) according to key terms from previous literature reviews. The complete search strategy is attached (appendix 1). The terms will be modified as necessary for other databases. We will not apply any language or date restrictions.
Study records			
Data management	11a	Page 6	Duplicates will be omitted using EndNote software (version X7.0). EndNote software will also be used to manage records. We will illustrate the selection process in a PRISMA diagram.
Selection process	11b	Page 6	Two review authors (WM and JS) will independently assess abstracts and titles of studies identified by literature search. Revelant studies will be selected against the predefined inclusion criteria. If necessary, reviewers will examine full-text reports to identify eligible studies. Any disagreement will be resolved by consensus.
Data collection process	11c	Page 6	Two review authors (YL and CZ) will independently extract data from the included studies. Calibration exercises will be conducted to ensure consistency across reviewers before starting the review. Where necessary we will contact study authors for clarification of data and additional information. Any disagreement will be resolved by consensus or consultation with a third review author.
Data items	12	Page 6	The following information will be extracted using a predetermined data form: general information (title, authors, country of study, funding, year of publication, registry number); details of study (aim, design, inclusion and exclusion criteria, method of randomization and allocation); study population (age, sex, sample size, number for analysis, type of stroke); intervention characteristics (type, duration, dose, follow-up time points, compliance); outcome (primary and secondary outcomes, time points, method of outcome assessments, blinding of outcome assessment).
Outcomes and prioritization	13	Page 5	The primary outcome measure will be frequency of bowel movement. Bowel movement frequency is defined as the mean number of spontaneous bowel movements per week.<br>Secondary outcome measures include proportion of patients with stool consistency, proportion of patients using rescue medication such as laxatives or rectal evacuants, Quality of life (QoL), and mean transit time.

For peer review only

			Stool consistency will be defined by trialists or measured based on scales such as Bristol Stool Form Scale (BSFS).<br>QoL will be measured by generic or condition-specific scales, such as Short Form 36 Health Surveys (SF-36).<br>Transit time is defined as the time from the first perception of wanting to defaecate to the finish of defaecation.<br>We will sum up the number of adverse events (AEs), and calculate the proportion of AEs.
Risk of bias in individual studies	14	Page 6-7	Two authors (HL and HS) will independently assess the risk of bias using the approach recommended by Cochrane Handbook for Systematic Reviews of Interventions. The following risk of bias domains will be assessed: sequence generation (selection bias); allocation concealment (selection bias); blinding of participants and personnel (performance bias); blinding of outcome assessment (detection bias); incomplete outcome data (attrition bias); selective outcome reporting (reporting bias); other bias.<br>We will attempt to describe what is reported to have happened in each study for each domain of risk of bias. Thus, we will be able to provide the rationale for the judgement of whether this domain is at low, high, or unclear risk of bias. Where necessary we will contact authors of included studies for missing information or clarification.<br>If all domians are at low risk of bias, the overall risk of bias of individual studies will be categorised as low risk of bias. Otherwise, overall risk of bias will categorised as high risk of bias. The 'Risk of bias' summary will be presented graphically.
Data synthesis	15a	Page 7	We will combine more than one trial to estimate pooled intervention effect using the meta-analysis when studies examine the same intervention and outcomes with comparable methods in similar populations.
	15b	Page 7-8	We will pool the continuous data using the inverse variance method, and dichotomous data using the Mantel-Haenszel method.<br>We will use the fixed-effect model to combine data when statistical heterogeneity is low (I^2 <

For peer review only - http://bmjopen.bmj.com/site/about/guidelines.xhtml

			50%). However, when $I^2 > 50\%$, the random-effect model will be used to provide a more conservative estimate of effect.<br>All analyses will be conducted with Review Manager 5.3 software.
	15c	Page 8	We will perform the following subgroup analyses to investigate heterogeneity when sufficient data are available. We will conduct subgroup analyses based on age, sex, type of stroke (hemorrhagic and ischemic stroke), different definitions of constipation, phase of stroke (acute, subacute or chronic), type of acupuncture (manual acupuncture, electroacupuncture, etc.), and type of control group (placebo, sham acupuncture, no treatment or another active treament or medication). A subgroup analysis based on population with different diet habbits is under plan considering that diet habits may play an important role in constipation development.<br>The intervention effect will be analyzed using the Chi^2 test, with $P < 0.05$ indicating statistically significant differences between subgroups.<br>We will perform sensitivity analyses to evaluate the robustness of the pooled results excluding trials with high risk of bias, and trials in which a prior history of constipation before the stroke diagnosis is not investigated, and the option of using missing data (worst-case scenario analysis, extreme worst-case analysis, or extreme best-case analysis).
	15d	Page 8	If a meta-analysis is not possible, we will provide a narrative summary of the results from individual studies.
Meta-bias(es)	16	Page 7	When a meta-analysis includes 10 or more RCTs, we will assess asymmetry using funnel plots visually. In addtition, we will test asymmetry using the Harbord modified test for dichotomous outcomes and Egger test for continuous outcomes.
Confidence in cumulative evidence	17	Page 8	We will prepare 'Summary of findings' tables including a grade of the overall quality of the body of evidence for each outcome using GRADEpro GDT.<br>Two review authors will independently assess the quality of the body of evidence according to five GRADE criteria: study limitations, imprecision, inconsistency, indirectness, and publication bias. It will fall into one of four possible ratings (high, moderate, low and very low). Any discrepancies

For peer review only

			will be resolved by consensus or consultation with a third review author.

For peer review only
